# HIV care-seeking behaviour after HIV self-testing among men who have sex with men in Beijing, China: a cross-sectional study

**DOI:** 10.1186/s40249-017-0326-y

**Published:** 2017-06-28

**Authors:** Xian-Long Ren, Zun-You Wu, Guo-Dong Mi, Jennifer M. McGoogan, Ke-Ming Rou, Yan Zhao, Nanci Zhang

**Affiliations:** 1 Division of Prevention Intervention, National Center for AIDS/STD Control and Prevention, Chinese Center for Disease Control and Prevention, 155 Changbai Road, Changping District, Beijing, 102206 People’s Republic of China; 2 Office of the Director, National Center for AIDS/STD Control and Prevention, Chinese Center for Disease Control and Prevention, 155 Changbai Road, Changping District, Beijing, 102206 People’s Republic of China; 3 Division of Treatment and Care, National Center for AIDS/STD Control and Prevention, Chinese Center for Disease Control and Prevention, 155 Changbai Road, Changping District, Beijing, 102206 People’s Republic of China

**Keywords:** Men who have sex with men, HIV self-testing, HIV care, Linkage to care, Care cascade, China

## Abstract

**Background:**

Men who have sex with men (MSM) has become the group with the fastest growing HIV epidemic in China. Since many Chinese MSM are conducting HIV self-testing, we aimed to determine the rate of HIV care seeking after self-testing, examine characteristics of “seekers” compared to “non-seekers,” and explore factors associated with HIV care-seeking behaviour.

**Methods:**

A cross-sectional study design was used and an online survey was conducted in Beijing, China in 2016, among users of a popular Chinese gay networking smart phone application. Chi-square test was used to compare characteristics of those who sought HIV care (“seekers”) and those who did not (“non-seekers”). Univariate and multivariate logistic regression analyses were conducted to assess factors associated with HIV care seeking.

**Results:**

Among 21,785 screened, 2383 participants (10.9%) were included in the study. A total of 380 participants (15.9%) reported seeking HIV care after HIV self-testing while 2003 (84.1%) did not. Lack of knowledge of the “window period” (adjusted odds ratio [A*OR*] = 0.68, 95% confidence interval [95% *CI*] = 0.47–0.97, *P* = 0.04) was associated with reduced odds of seeking HIV care, while lower monthly income (A*OR* = 1.29, 95% *CI* = 1.03–1.62, *P* = 0.03) and obtaining HIV self-testing kits from health facilities (A*OR* = 2.40, 95% *CI* = 1.81–3.17, *P* < 0.001), and non-governmental organizations (A*OR* = 2.44, 95% *CI* = 1.79–3.34, *P* < 0.001) was associated with increased odds of seeking HIV care. Among those who sought HIV care, a large majority (92.4%) had non-reactive HIV self-testing results. Only 29 out of 265 with reactive, uncertain, or unknown results sought HIV care.

**Conclusions:**

We found a very low rate of HIV care seeking among our sample of urban Chinese MSM. The observation that most with reactive, uncertain, or unknown results did not seek HIV care is a cause for concern. These people should be paid more attention and helped to enter the care cascade. Our findings highlight that interventions aimed at improving linkage to care after HIV self-testing are urgently needed. However, further study is required to inform the design and implementation of future interventions aiming to encourage HIV care-seeking behaviour.

**Electronic supplementary material:**

The online version of this article (doi:10.1186/s40249-017-0326-y) contains supplementary material, which is available to authorized users.

## Multilingual abstracts

Please see Additional file 1 for translations of the abstract into the five official working languages of the United Nations

## Background

Testing for human immunodeficiency virus (HIV) infection is an important strategy for prevention and control the global epidemic as evidenced by its inclusion as the first goal of the Joint United Nations Programme on HIV/AIDS (UNAIDS) 90-90-90 targets—90% of people living with HIV (PLHIV) diagnosed by the year 2020 [1]. However, approximately 43% of PLHIV worldwide were still unaware of their infection status in 2015 [2]. In China, there were estimated 850,000 PLHIV in 2015, but approximately 35% of them remained unidentified [3]. Sexual contact has become the most common mode of transmission and men who have sex with men (MSM) has become the high-risk group with the most rapidly rising HIV prevalence, climbing from approximately 1.5% in 2005, to 8.0% in 2015 [4, 5].

HIV voluntary counselling and testing (VCT) services have been free since 2002, and accessible nationwide since 2004, and provider-initiated testing and counselling (PITC), although not free, has been available since 2007 [6]. Moreover, a variety of intensive education efforts focused on promoting testing uptake have been ongoing since 2003 [6], and MSM-friendly community-based organizations have had gradually increasing involvement in testing mobilization campaigns [5, 7–9]. Nevertheless, testing rates remain persistently low among Chinese MSM. A meta-analysis in 2012 found a rate of lifetime HIV testing of 47% and testing in the past 12 months of only 38% [10].

Officially, HIV testing in China must be performed by trained medical personnel only and is purely facility-based, being offered primarily at VCT sites, hospitals and specialized HIV/AIDS clinics, and at Centers for Disease Control and Prevention (CDC) offices. [11] However, a recent study found willingness to test at CDCs among only 28% of Chinese MSM participants [12]. Barriers to traditional, facility based testing among Chinese MSM primarily include inconvenience (facilities are too far away or days/hours of operation conflict with work), worry about privacy and confidentiality, fear of social stigma and resulting discrimination, and unwillingness to register for testing under one’s real name [5, 12–14].

HIV self-testing may help to reach populations that do not access conventional facility-based testing services [2, 5, 15–18], having advantages including convenience and flexibility, and privacy and confidentiality. Despite official policy, a wide range of HIV self-test kit products have become readily available in China both online and at medical facilities. The present study aimed to determine the rate of HIV care seeking after self-testing, examine characteristics of those who sought HIV care after self-testing, and explore factors associated with HIV care seeking.

## Methods

### Study design and setting

**Fig. 1:**
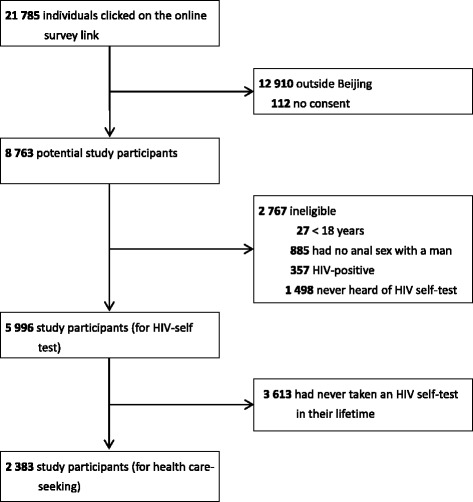
Study design and population

A cross-sectional study design was used, see Fig. 1, and an online survey among MSM in Beijing was conducted from May 14 to May 17, 2016. The results of a portion of this survey have been published elsewhere [19], and thus some details of survey-specific methods are not repeated here.

For the purposes of this study, “HIV self-testing” was defined as using an HIV test on oneself without the supervision, consultation, or counselling of trained healthcare staff. “HIV care seeking” was defined approaching health workers at CDC, clinic/hospital, or non-governmental organization (NGO) venues, after performing HIV self-testing, to obtain further HIV counselling, testing, or other care services.

### Eligibility criteria

Participants were eligible for the study if they met the following criteria: (1) being users of a popular gay networking smart phone application (App) who registered in the App with Beijing location and had smart phone global positioning satellite (GPS) signals localized within Beijing, (2) being male and having ever had anal sex with men, (3) being ≥18 years of age, (4) believing one’s HIV serostatus to be negative, or not knowing one’s HIV serostatus, (5) having ever taken an HIV self-test (i.e., had ever used of an HIV self-test kit on oneself without professional consultation), and (6) having provided informed consent.

### Data collection and quality assurance

The questionnaire was adapted from that of a recent study by Wong and colleagues [20]. It was modified for the present study and then piloted among 110 MSM selected by convenience sampling methods from QQ, a popular Chinese instant messaging software service. For the face-to-face pilot, a total of 56 MSM were asked to complete the questionnaire on paper and then give feedback on how to improve it. For the online pilot, a total of 110 MSM were asked to complete an online version of the questionnaire. After analysing the results, several small technical errors within the questionnaire were identified. The questionnaire was further modified based on feedback received and errors identified during the pilot. Participants included in the pilots were excluded from the study.

Each participant was given a unique identifier based on his internet protocol (IP) address and was only allowed to take the survey once. Participants were provided with definitions of the terms “HIV self-testing” and “HIV care-seeking.” The final questionnaire collected information on demographics, sexual behaviour, drug use, HIV self-testing experience, and sexual health history. The questionnaire was adaptive, only participants who answered affirmatively to certain questions were presented additional questions on that topic.

Questions were displayed one at a time and participants were to select from a list of answer choices provided. For nearly all questions, participants were only allowed to select one answer. There were two questions for which participants could choose multiple answers. Those questions were: “How do you find information on HIV self-testing?” and “What factors are most important when choosing an HIV self-test kit?”

### Statistical analysis

Characteristics of all participants were described by categorical variables presented as number and percent. Participants were separated into two subgroups according to whether they sought HIV care after self-testing (“seekers”), or not (“non-seekers”). Differences between subgroups were assessed by Chi-square analysis and *P*-values are presented.

Univariate and multivariate logistic regression modelling was used to explore factors associated with seeking HIV care after HIV self-testing. HIV care seeking was given a value of 1 and no HIV care seeking was given a value of 0. Independent variables were selected based on results of univariate analysis, all variables with outcomes of *P* < 0.05 were entered into the multivariate model. *P*-values <0.05 were considered statistically significant. All confidence intervals presented were 95% confidence intervals (95% *CI*). All analyses were performed using Version 18.0 SPSS software Version 18.0 (Statistical Package for the Social Science, IBM, New York, USA).

### Ethics

The study protocol was reviewed and approved by the Institutional Review Board (IRB) of the National Center for AIDS/STD Control and Prevention, Chinese Center for Disease Control and Prevention. Informed consent was collected electronically for all study participants. The survey was anonymous and no compensation for participation was provided.

## Results

### The study population

A total 21,785 users who clicked on the online survey link were screened for study inclusion. Among them, 12,910 were outside Beijing, 885 had never had anal sex with a male partner, 27 were <18 years old, 5111 had never taken an HIV self-test in their lifetime, 357 had a known HIV-positive serostatus, and 112 did not provide informed consent. Therefore, the final study population was 2383 MSM (of 21,785, 10.9%), see Fig. 1.

### Characteristics of participants

Among study participants, 62.6% were <30 years of age, 79.1% had college-level education or greater, 71.6% had lived in Beijing for at least 2 years, 86.9% were unmarried, and 74.5% self-identified as homosexual, see Table 1. A majority reported having had fewer than 2 male sex partners (58.6%) and consistently using condoms (62.0%) during anal sex with male partners in past 3 months. Furthermore, 45.4% reported having ever used drugs in their lifetimes.

**Table 1:** Characteristics of all participants by HIV care-seeking status^a^

Variable	All Participants [*N* = 2383] *n* (%)	“Seekers” Subgroup [*n* = 380] *n* (%)	“Non-Seekers” Subgroup [*n* = 2003] *n* (%)	*P*-value
*Demographic Characteristics*				
Age (years)				0.48
18–24	649 (27.2)	106 (27.9)	543 (27.1)	
25–29	841 (35.3)	124 (32.6)	717 (35.8)	
≥ 30	893 (37.5)	150 (39.5)	743 (37.1)	
Education level				0.03
≤ High School^b^	499 (20.9)	95 (25.0)	404 (20.2)	
≥ College and above	1884 (79.1)	285 (75.0)	1599 (79.8)	
Monthly income (CNY)				0.03
≤ 5000	997 (41.8)	178 (46.8)	819 (40.9)	
> 5000	1386 (58.2)	202 (53.2)	1184 (59.1)	
Duration of residence in Beijing (years)				0.98
< 2	676 (28.4)	108 (28.4)	568 (28.4)	
≥ 2	1707 (71.6)	272 (71.6)	1435 (71.6)	
Marital status				0.63
Married	313 (13.1)	47 (12.4)	266 (13.3)	
Unmarried	2070 (86.9)	333 (87.6)	1737 (86.7)	
Sexual orientation				0.37
Homosexual	1775 (74.5)	293 (77.1)	1482 (74.0)	
Bisexual	604 (25.3)	86 (22.6)	518 (25.9)	
Unknown/Unsure	4 (0.2)	1 (0.3)	3 (0.1)	
*Sexual Behaviour Characteristics*				
Number of male anal sex partners in past 3 months				0.07
< 2	1397 (58.6)	239 (62.9)	1158 (57.8)	
≥ 2	986 (41.4)	141 (37.1)	845 (42.2)	
Condom use with male anal sex partners in past 3 months				0.86
Always	1105 (62.0)	171 (61.5)	934 (62.1)	
Inconsistent	678 (38.0)	107 (38.5)	571 (37.9)	
Missing	600			
*Drug Use Behaviour Characteristics*				
Drug use ever in lifetime				0.95
Yes	1082 (45.4)	172 (45.3)	910 (45.4)	
No	1301 (54.6)	208 (54.7)	1093 (54.6)	
Anal sex after drug use				0.70
Yes	927 (38.9)	149 (86.6)	778 (85.5)	
No	155 (6.5)	23 (13.4)	132 (14.5)	
No drug use	1301 (54.6)			
Condom use during anal sex after drug use				0.92
Always	613 (25.7)	98 (65.8)	515 (66.2)	
Inconsistent	314 (13.2)	51 (34.2)	263 (33.8)	
No anal sex after drug use	155 (6.5)			
No drug use	1301 (54.6)			
*Use of HIV Self-Test Kits*				
Source of HIV self-test kits				< 0.001
Internet	1448 (60.8)	182 (47.9)	1266 (63.2)	
Health facilities	422 (17.7)	102 (26.8)	320 (16.0)	
NGOs	285 (12.0)	73 (19.2)	212 (10.6)	
Friends/Other	228 (9.6)	23 (6.1)	205 (10.2)	
Reason for most recent testing				0.14
Routine	1154 (48.4)	202 (53.2)	952 (47.5)	
Had unprotected anal sex	469 (19.7)	76 (20.0)	393 (19.6)	
Had physical discomfort	165 (6.9)	26 (6.8)	139 (6.9)	
Going to have a new/lifetime partner	369 (15.5)	45 (11.8)	324 (16.2)	
To confirm a previous result	129 (5.4)	21 (5.5)	108 (5.4)	
Other	97 (4.1)	10 (2.6)	87 (4.3)	
Considered the “window period” in deciding when to test^c^				0.02
Yes	1584 (66.5)	269 (70.8)	1315 (65.7)	
No	426 (17.9)	70 (18.4)	356 (17.8)	
Don’t know	373 (15.7)	41 (10.8)	332 (16.6)	
Most recent HIV self-test result				< 0.001
Non-reactive	2118 (88.9)	351 (92.4)	1767 (88.2)	
Reactive	30 (1.3)	5 (1.3)	25 (1.2)	
Uncertain	92 (3.9)	24 (6.3)	68 (3.4)	
Unknown	143 (6.0)	0 (0.0)	143 (7.1)	
Sought healthcare for STI symptoms in past 1 year				0.27
Yes	245 (10.3)	45 (11.8)	200 (10.0)	
No	2138 (89.7)	335 (88.2)	1803 (90.0)	

*HIV* human immunodeficiency virus, *CNY* Chinese Yuan, *CDC* Centers for Disease Control, *NGO* non-government organization, *STI* sexually-transmitted infection

^a^HIV care seeking was defined as an individual approaching health workers at clinic, hospital, NGO, or CDC venues for the purpose of obtaining further HIV counselling, testing, or other care services, after administering an HIV self-test

^b^The category “High School” includes also vocational or technical school

^c^For the most recent self-test taken

Compared to “non-seekers,” a smaller proportion of “seekers” had college or higher levels of education (75.0% versus 79.8%, *P* = 0.03) and monthly incomes of >5000 Chinese Yuan (CNY, > approximately 733 US Dollars, 53.2% versus 59.1%, *P* = 0.03). A larger proportion of “seekers” considered the “window period” in deciding when to take their most recent HIV self-test (70.8% versus 65.7%, *P* = 0.02). In terms of participants’ most recent HIV self-test result, a greater proportion of “seekers” had a negative result (92.4% versus 88.2%, *P* < 0.001), see Table 1

### Factors associated with HIV care-seeking behaviour

Income of 5000 CNY or less per month was associated with increased odds of HIV care-seeking behaviour (adjusted odds ratio [A*OR*] = 1.29, 95% *CI* = 1.03–1.62, *P* = 0.03), obtaining HIV self-testing kits from health facilities (A*OR* = 2.40, 95% *CI* = 1.81–3.17, *P* < 0.001), and NGOs (A*OR* = 2.44, 95% *CI* = 1.79–3.34, *P* < 0.001), was associated with increased odds of HIV care-seeking behaviour. Finally, answering “Don’t Know” to the question “Did you consider the window period in deciding when to take your most recent HIV self-test?” was associated with decreased odds of HIV care-seeking behaviour (A*OR* = 0.68, 95% *CI* = 0.47–0.98, *P* = 0.04). Those who were not sure their HIV self-test result were more likely to have HIV care-seeking behaviour (A*OR* = 1.60, 95% *CI* = 0.97–2.63, *P* = 0.07), see Table 2.

**Table 2:** Factors associated with HIV care-seeking behaviour after HIV self-testing

Variable^a^	*OR* (95% *CI*)	*P*-value	A*OR* (95% *CI*)	*P*-value
*Demographic Characteristics*				
Age (years)				
18–24	1.00			
25–29	0.89 (0.67–1.18)	0.40		
≥ 30	1.03 (0.79–1.36)	0.81		
Education level				
≥ College and above	1.00		-	
≤ High School ^b^	1.32 (1.02–1.71)	0.03	-	-
Monthly income (CNY)				
> 5000	1.00		1.00	
≤ 5000	1.27 (1.02–1.59)	0.03	1.29 (1.03–1.62)	0.03
Duration of residence in Beijing (years)				
< 2	1.00			
≥ 2	0.99 (0.78–1.27)	0.98		
Marital status				
Married	1.00			
Unmarried	1.09 (0.78–1.51)	0.63		
Sexual orientation				
Homosexual	1.00			
Bisexual	0.84 (0.65–1.09)	0.19		
Unknown/Unsure	1.69 (0.18–16.27)	0.65		
*Sexual Behaviour Characteristics*				
Number of male anal sex partners in past 3 months				
< 2	1.00			
≥ 2	0.81 (0.65–1.01)	0.07		
Condom use with male anal sex partners in past 3 months				
Always	1.00			
Inconsistent	1.02 (0.79–1.33)	0.86		
*Drug Use Behaviour Characteristics*				
Drug use ever in lifetime				
No	1.00			
Yes	0.99 (0.80–1.24)	0.95		
Anal sex after drug Use				
No	1.00			
Yes	1.10 (0.68–1.77)	0.70		
Condom use during anal sex after drug use				
Always	1.00			
Inconsistent	1.02 (0.70–1.48)	0.92		
*Use of HIV Self-Test Kits*				
Source of HIV self-test kits				
Internet	1.00		1.00	
Health facilities	2.22 (1.69–2.91)	< 0.001	2.40 (1.81–3.17)	< 0.001
NGOs	2.40 (1.76–3.26)	< 0.001	2.44 (1.79–3.34)	< 0.001
Friends/Other	0.78 (0.49–1.23)	0.29	0.87 (0.55–1.38)	0.56
Reason for most recent testing				
Routine	1.00			
Had unprotected anal sex	0.91 (0.68–1.22)	0.53		
Had physical discomfort	0.88 (0.57–1.38)	0.58		
Going to have a new/lifetime partner	0.66 (0.46–0.93)	0.02		
To confirm a previous result	0.92 (0.56–1.50)	0.73		
Other	0.54 (0.27–1.06)	0.54		
Did you consider the “window period” in deciding when to take your most recent HIV self-test?				
Yes	1.00		1.00	
No	0.96 (0.72–1.28)	0.79	0.89 (0.66–1.19)	0.43
Don’t know	0.60 (0.43–0.86)	0.005	0.68 (0.47–0.97)	0.04
Most recent HIV self-test result				
Non-reactive	1.00		1.00	
Active	1.01 (0.38–2.65)	0.99	0.95 (0.35–2.56)	0.92
Uncertain	1.78 (1.10–2.87)	0.02	1.60 (0.97–2.63)	0.07
Unknown				
Sought healthcare for STI symptoms in past 1 year				
No	1.00			
Yes	1.21 (0.86–1.71)	0.28		

*HIV* human immunodeficiency virus, *OR* odds ratio, *95% CI* 95% confidence interval, A*OR* adjusted odds ratio, *CNY* Chinese Yuan, *CDC* Centers for Disease Control, *NGO* non-government organization, *STI* sexually-transmitted infection

^a^ Only variables having results found to be statistically significant in univariate and multivariate analyses are included.

^b^ The category “High School” includes also vocational or technical school.

Among the 30 participants who reported reactive HIV self-test results, only 5 (16.7%) sought HIV care, and no differences were found in sexual or drug using behaviours between “seekers” and “non-seekers,” see Table 3.

**Table 3:** Sexual behaviour characteristics of participants who had reactive results on their most recent HIV self-test

Variable	All Participants [*N* = 30] *n* (%)	“Seekers” Subgroup [*n* = 5] *n* (%)	“Non-Seekers” Subgroup [*n* = 25] *n* (%)	*P*-value
*Sexual Behaviour Characteristics*				
Number of male anal sex partners in past 3 months				0.57
< 2	14 (46.7)	2 (40.0)	12 (48.0)	
≥ 2	16 (53.3)	3 (60.0)	13 (52.0)	
Condom use with male anal sex partners in past 3 months				0.36
Always	13 (54.2)	3 (75.0)	10 (50.0)	
Inconsistent	11 (45.8)	1 (25.0)	10 (50.0)	
Missing	6			
*Drug Use Behaviour Characteristics*				
Drug use ever in lifetime				0.68
Yes	18 (60.0)	3 (60.0)	15 (60.0)	
No	12 (40.0)	2 (40.0)	10 (40.0)	
Anal sex after drug use				0.69
Yes	16 (88.9)	3 (100.0)	13 (86.7)	
No	2 (11.1)	0 (0.0)	2 (13.3)	
No Drug Use	12			
Condom use during anal sex after drug use				0.21
Always	10 (62.5)	3 (100.0)	7 (53.8)	
Inconsistent	6 (37.5)	0 (0.0)	6 (46.2)	
No anal sex after drug use	2			
No drug use	12			
Sought healthcare for STI symptoms in past 1 year				0.30
Yes	6 (20.0)	0 (0.0)	6 (24.0)	
No	24 (80.0)	5 (100.0)	19 (76.0)	

*HIV* human immunodeficiency virus, *STI* sexually-transmitted infection

## Discussion

As expected, we found a very low rate of HIV care seeking after HIV self-testing—only 15.9% among our MSM sample. These “seekers” tended to have lower educational attainment and lower monthly incomes, but were more conscious of the window period in deciding when to self-test and tended to source their kits health facilities (e.g., CDC offices, hospitals, NGOs). Factors with statistically significant associations with seeking HIV care after self-testing included having lower incomes and sourcing kits at health facilities.

Although a recent systemic review and meta-analysis found that data on linkage to care after HIV self-testing was lacking, a few studies other low- and middle-income countries support our finding of low rates of care seeking after HIV self-testing and offer some ideas on how linkage may be improved [21]. Two studies with linkage to care outcomes among male partners of pregnant women in Kenya have found that although 72% reportedly sought confirmatory testing after HIV self-testing, only 2 of the 8 men who had a reactive self-test result were linked to care [22, 23] . A small study in Peru in which 147 MSM and 45 transgender women were interviewed found that although 82% were willing to use HIV self-test kits, only 55% reported that they would seek confirmatory testing after a reactive result [24] . A qualitative study in South Africa identified many perceived barriers to linkage to HIV care after self-testing, which has informed a pilot intervention there aimed at improving post-self-test linkage via trained counsellors reaching out to testers via mobile phone technologies to encourage and measure HIV care seeking [25]. A trial of in-home versus facility-based linkage to care and ART initiation after HIV self-testing in Malawi found a significant improvement in ART initiation rates with the in-home linkage model [26]. Finally, a study of linkage to care among HIV self-testing Chinese MSM found that social entrepreneurship testing model resulted in all 8 individuals in the study confirmed to have HIV infection being successfully linked to care [27].

Notably, we found that a large majority of those who sought HIV care did so after a non-reactive (negative) self-test result (92.4%). Moreover, a majority of participants who self-reported that their most recent HIV self-test result was either reactive (positive), uncertain, or unknown did not seek HIV care afterward. Unfortunately, participants were not asked their reasons for seeking (or not seeking) HIV care after self-testing, and the cross-sectional study design does not allow examinations of causality. Thus, we were unable to explore the motives of seekers and non-seekers. Nevertheless, we can suggest some possible explanations that warrant future study.

Firstly, we found that participants who obtained their kits from health facilities were more than two times more likely to later seek HIV care after using their self-test kit. It is possible that participants received face-to-face counselling about self-testing and subsequent linkage to care while at these facilities. Point-of-sale pre-test counselling was, in fact, included as a recommended minimum standard of service delivery for HIV self-testing programs by the WHO in 2013. Expanding and strengthening point-of-sale pre-test counselling may be one way to improve linkage to HIV care for those who choose to self-test.

Secondly, we also found that participants who responded “don’t know” when asked if they considered the window period in deciding when to test were half as likely to later seek HIV care after using their self-test kit. It is possible that those with perhaps relatively higher HIV/AIDS knowledge may seek HIV care after a non-reactive self-test result because of a lack of trust in the quality of the product and/or the reliability of the result. Globally, quality concerns about self-test kits sold over-the-counter have been expressed not only by self-test takers, but also by public health experts and policymakers [18]. In China, and specifically among MSM, several studies have found accuracy of HIV self-test kits to be a major concern [16, 17, 20, 28–30]. This lack of trust may be severely limiting the HIV self-testing uptake, and perhaps subsequent linkage to HIV care.

The findings of a nearly 40% rate of HIV self-testing uptake and over 90% willingness to use HIV self-test kits in the future in this same population are encouraging [19]. However, further study on HIV care-seeking behaviour and features of HIV care services that could improve its uptake by this population is required. Outcomes of these studies could possibly inform the design of future education and self-testing promotion campaigns, community-based self-testing mobilization efforts, point-of-sale pre-test counselling, and other interventions may help to drive self-testing uptake and subsequent linkage to HIV care for those who obtain reactive, uncertain, or unknown results from self-test kit use. However, all of these must first be preceded by regulation and policy. The China Food and Drug Administration must, itself, review the evidence for safety and reliability of HIV self-test products, conduct its own risk-benefit analysis based on the China context, examine test systems as a whole, including instructions for use in the hands of inexperienced users, and partner with manufacturers to ensure products meet acceptable standards, receive approval, and are regularly audited against quality standards. Furthermore, China’s public health policymakers must create a framework for inclusion of HIV self-testing in existing programming, drive education, and encourage community-based test mobilization, not just for MSM, but for all high-risk populations [2, 18, 29]. This important new tool in the fight against HIV/AIDS must be brought to bear such that it can reach its potential and bring China closer to the UNAIDS 90-90-90 goals.

## Limitations

This study had several limitations. Firstly, participants were all users of a popular gay networking App, and therefore MSM who were not users of the App could not be recruited. Since it is possible that some subgroups were not represented (e.g., those who do not have smart phones, or use gay networking Apps), the results of this study may not be generalizable to the entire Chinese MSM population. Secondly, because of severe dual-stigma facing MSM in China who have HIV infection, self-reported responses to questions related to HIV self-test results, in particular, may be subject to social desirability bias. Thirdly, questions as to the reasons for seeking or not seeking healthcare after HIV self-testing were not asked of participants as a part of the survey’s questionnaire and because of the retrospective, cross-sectional study design, it is not possible to determine causality.

## Conclusions

Our finding of a low rate of HIV care seeking after HIV self-testing (15.9%), and the even lower rate among those who obtained reactive, uncertain, or unknown results (10.9%) is very concerning. HIV self-testing could be an important key to growing the presently sub-optimal testing uptake of MSM in China [5, 16, 17, 29–32], and helping China reach the UNAIDS 90-90-90 targets [1]. However, dramatic improvements in linkage to further HIV testing and care for HIV self-testers is of critical importance. Although further study is required to inform the design and implementation of interventions aimed to close this gap, China must take urgent action to help those who screen reactive on HIV self-tests to get the care they need.

## Additional file

Additional file 1: Multilingual abstracts in the five official working languages of the United Nations. (PDF 378 kb)

## Abbreviations

95% *CI*: 95% confidence interval
A*OR*: Adjusted odds ratio
App: Smart phone application
CDC: Center for Disease Control and Prevention
CNY: Chinese Yuan
GPS: Global positioning satellite
HIV: Human immunodeficiency virus
IP: Internet protocol
IRB: Institutional Review Board
MSM: Men who have sex with men
NGO: Non-governmental organization
*OR*: Odds ratio
PITC: Provider-initiated testing and counselling
PLHIV: People living with HIV
QQ: An instant messenger developed by the Tencent company
SPSS: Statistical package for the social sciences
STI: Sexually-transmitted infection
UNAIDS: The Joint United Nations Programme on HIV/AIDS
USD: United States Dollar
VCT: Voluntary counselling and testing
WHO: World Health Organization
