# Supplementary material for: HIV care-seeking behaviour after HIV self-testing among men who have sex with men in Beijing, China: a cross-sectional study
Source: Infect Dis Poverty. 2017 Jun 28;6:112. doi: 10.1186/s40249-017-0326-y (PMC5488343; doi:10.1186/s40249-017-0326-y)
Supplement: Additional file 1: — Multilingual abstracts in the five official working languages of the United Nations. (PDF 378 kb) [file 40249_2017_326_MOESM1_ESM.pdf]

Translation of the abstract into the five official working languages of the United Nations

السلوك الساعي إلى العناية بفيروس نقص المناعة البشرية بعد الفحص الذاتي لفيروس نقص المناعة البشرية بين الرجال المثليين في ببكين، الصين: دراسة شامله لعده قطاعات

شيان--لونغ رن، سون-يو وو، قوه-دونغ مي، جنيفر م. ماكغوغان، كي-مينغ رو، يان تشاو ونان-تسي تشانغ

ملخص

الخلفية: أصبحت مجموعة الرجال الذين يمارسون الجنس مع رجال مثلهم (المثليين الجنس) هي المجموعة التي تشهد أسرع إنتشارا لوباء لفيروس نقص المناعة البشرية في الصين وبما ان العديد من المثليين الصينيين يجرون فحصا ذاتيا لفيروس نقص المناعة البشرية، فاننا هدفنا لتحديد معدل السعي لطلب العناية بفيروس نقص المناعة البشرية بعد الفحص الذاتي، ودراسة خصائص "الساعيين" بالمقارنة مع "غير الساعيين" واستكشاف العوامل المرتبطة بسلوك طلب العناية بفيروس نقص المناعة البشرية.

الأساليب: تم تصميم دراسة متعددة القطاعات وتم إستخدامها كما أجريت دراسة استقصائية علي الإنترنت في بكين بالصين في ٢٠١٦ بين مستخدمي تطبيق شبكة المثليين الصيبيين الشائع علي الهواتف الذكية وتم استخدم اختبار "خي مربع" لمقارنه خصائص أولئك الذين يسعون إلى الحصول علي الرعاية الخاصة بفيروس نقص المناعة البشرية ("الساعيين") وأولئك الذين لا يسعون ("غير الساعيين"). وأجريت تحليلات الانحدار اللوجستي ذو المتغير الواحد والمتعدد المتغيرات لتقييم العوامل المرتبطة بالعناية بفيروس نقص المناعة البشرية.

النتائج: من بين العينات ال ٢١٧٨٥ التي تم فرزها تم إدراج عدد ٢٣٨٣ (١٠,٩٪) مشتركا في هذه الدراسة وابلغ ٣٨٠ مشاركا (١٥,٩٪) عن التماس الرعاية الخاصة بفيروس نقص المناعة البشرية بعد الفحص الذاتي للفيروس بينما لم يبلغ ٢٠٠٣ مشاركا (٨٤,١٪) وكان الافتقار إلى معرفه "فتره الإطار" (نسبه الارجحيه المعدلة [AOR] = ٠,٨٦، ٩٥٪ فاصل ثقة [٩٥٪ حدود ثقة] = ٠,٤٧ – ٠,٩٧، P= ٠,٠٤ مرتبطا بانخفاض احتمالات التماس الرعاية المتعلقة بفيروس نقص المناعة البشرية في حين ان الدخل الشهري المنخفض (AOR= ١,٢٩، ٩٥٪ حدود ثقة = ١,٠٣ – ١,٦٢، P= ٠,٠٣) والحصول علي مجموعات الفحص الذاتي لفيروس نقص المناعة البشرية من المرافق الصحية (AOR= ٢,٤٠، ٩٥٪ حدود ثقة = ١,٨١ – ٣,١٧، P< ٠,٠٠١) والمنظمات غير الحكومية (AOR= ٢,٤٤، ٩٥٪ حدود ثقة = ١,٧٩ – ٣,٣٤، P< ٠,٠٠١). كانوا مرتبطين بزيادة احتمالات التماس الرعاية المتعلقة بفيروس نقص المناعة البشرية ومن بين الذين التمسوا الرعاية الخاصة بفيروس نقص المناعة البشرية، كان للاغلبيه الكبيرة (٩٢,٤٪) نتائج غير متفاعلة للفحص الذاتي للفيروس ولم يسع سوي ٢٩ من ٢٦٥ الذين كانت لهم نتائج تفاعليه أو غير مؤكده أو غير معروفه إلى الحصول علي الرعاية الخاصة بفيروس نقص المناعة البشرية.

الاستنتاجات: وجدنا معدلا منخفضا جدا من الباحثين عن الرعاية الخاصة بفيروس نقص المناعة البشرية بين العينه التي لدينا من مثليين الجنس الصينيين من سكان الحضر وينبغي ان يولي هؤلاء الناس مزيدا من الاهتمام وان يتم مساعدتهم علي الدخول في سلسله الرعاية وتبرز النتائج التي توصلنا اليها ان هناك حاجة ماسة للتدخلات الرامية إلى تحسين الصلة بالرعاية بعد اجراء الفحص الذاتي لفيروس نقص المناعة البشرية غير انه يلزم اجراء مزيد من الدراسات للاسترشاد بها في تصميم وتنفيذ التدخلات المقبلة الرامية إلى تشجيع السلوك الساعي إلى العناية بفيروس نقص المناعة البشرية.

Translated from English version into Arabic by Mohamed Habib

北京市男男性行为者 HIV 自我检测后求医行为横断面研究

任仙龙，吴尊友，米国栋，Jennifer M. McGoogan, 柔克明，赵燕，Nanci Zhang

摘要
背景: 男男性行为者 (MSM) 已经成为中国艾滋病流行增长速度最快的人群。有很多 MSM 进行 HIV 自我检测。我们旨在研究 HIV 自我检测后的求医比例，分析“求医者”与“非求医者”之间特征区别，探索求医行为的影响因素。
方法: 采用横断面研究设计，2016 年对男同手机应用软件的北京用户开展网络在线调查。采用卡方检验比较“求医者”与“非求医者”的特征区别。采用单因素和多因素 logistic 回归分析方法，探索求医行为的影响因素。
结果: 在 21 785 名点击连接的人员中, 2 383 (10.9%)人被招募进入研究。其中，380 (15.9%)人报告在 HIV 自我检测后有求医行为，但 2 003 (84.1%) 人没有求医行为。对“窗口期”不了解 (调整比值比[A*OR*] = 0.68, 95% 可信区间[95% *CI*] = 0.47 – 0.97, *P* = 0.04) 是求医行为的阻碍因素。而每月低收入 (A*OR* = 1.29, 95% *CI* = 1.03 – 1.62, *P* = 0.03)、从卫生机构获得 HIV 自我检测试剂盒 (A*OR* = 2.40, 95% *CI*=1.81 – 3.17, *P* < 0.001)、或从民间组织获得 HIV 自我检测试剂盒 (A*OR*=2.44, 95% *CI* = 1.79 – 3.34, *P* < 0.001) 则是求医行为的促进因素。在求医者中，绝大部分 (92.4%) 为 HIV 自我检测结果阴性。在 265 名 HIV 自我检测为阳性、不确定或不清楚者中，仅 29 人前来求医。
结论: 我们发现 MSM 人群 HIV 自我检测后求医比例很低。我们观察到 HIV 自我检测结果为阳性、不确定或不清楚者未能求医的想象令人担忧。这些人则需要更多关注，以便帮助他们进入到医疗服务。我们的研究结果提示，干预工作需要改进 HIV 自我检测与医疗服务的衔接。未来的科学研究需要设计并实施旨在鼓励 HIV 求医行为。

Translated from English version into Chinese by Xian-Long Ren

Attitude après auto-dépistage du VIH des hommes ayant des relations sexuelles avec d'autres hommes en quête de soins à Beijing, Chine: une étude transversale

Xian-Long Ren, Zun-You Wu, Guo-Dong Mi, Jennifer M. McGoogan, Ke-Ming Rou[1], Yan Zhao and Nan-Ci Zhang

Résumé
Contexte: Le phénomène des hommes ayant des relations sexuelles avec d’autres hommes (HSH) est devenu la source de croissance la plus rapide de l’épidémie du VIH en Chine. Depuis que de nombreux Chinois HSM ont entrepris l’auto-dépistage du VIH, nous avions pour objectif de déterminer le taux de damande de soins pour le VIH après auto-diagnostic, d'examiner les caractéristiques des ‘réquerants’ par rapport aux ‘non- réquerants’, et d’explorer les facteurs liés à la demande de soins pour le VIH.
Méthodes: Une étude transversale a été utilisée et un sondage en ligne a été mis en place à Beijing, Chine, en 2016, parmi les utilisateurs d’une application populaire de smartphone du réseau de gays Chinois. Le test du chi carré a été utilisé pour comparer les caractéristiques de ceux qui sont en quête de soins pour le VIH (‘réquerants’) et de ceux qui ne l’en sont pas (‘non-réquerants’). Des analyses de régression logistique univariées et multivariées ont été menées afin d’évaluer les facteurs associés à la demande de soins pour le VIH.
Résultats: Parmi 21 785 personnes examinées, 2 383 participants (10,9%) ont été inclus dans l'étude. Un total de 380 participants (15,9%) ont déclaré avoir demandé de soins pour le VIH

après auto-dépistage, alors que 2 003 (84,1%) n'en ont pas demandé. Le manque de connaissances de la ‘période fenêtre’ (quotients de probabilités ajustés [a*OR*s] = 0,68, intervalles de confiance [*CIs*] à 95% [*CIs* 95%] = 0,47 – 0,97, p = 0,04) était associé à une réduction de la demande de soins pour le VIH, tandis que le faible revenu mensuel ([a*OR*s] = 1,29, [*CIs* 95%] = 1,03 – 1,62, p = 0,03) avec le fait d’obtenir des kits d’auto-dépistage du VIH auprès des centres de santé ([a*OR*s] = 2,40, [*CIs* 95%] = 1,81 – 3,17, p < 0001) et des organisations non gouvernementales ([a*OR*s] = 2,44; [*CI*s 95%] = 1,79 – 3,34, p < 0001) était associé au besoin accru d'avoir des soins pour le VIH. Parmi ceux qui étaient à la recherche de soins pour le VIH, une large majorité (92,4%) avait des résultats non-réactifs à l’auto-dépistage du VIH. Seulement 29 des 265 qui avaient des résultats réactifs, incertains ou inconnus en demandaient.

Conclusions: Notre étude révèle un très faible taux de demande de soins pour le VIH parmi notre échantillon de Chinois urbains HSH. L'observation selon laquelle la majorité ayant des résultats réactifs, incertains ou inconnus ne cherchait pas à avoir de soins pour le VIH est une cause préoccupante. Une attention particulière doit être portée à ces personnes et les aider à emboîter le pas de la damande de soins. Nos résultats soulignent que des interventions visant à améliorer le lien aux soins après auto-dépistage du VIH sont d'urgence. Toutefois, une étude plus approfondie est nécessaire afin d’éclairer sur la conception et la mise en œuvre des interventions futures visant à encourager la demande de soins pour le VIH.

Translated from English version into French by Kokouvi Kassegne

Поведение ухода за выздоравливающим ВИЧ после самостоятельного тестирования на ВИЧ среди мужчин, имеющих половые контакты с мужчинами в Пекине Китая: перекрестное исследование

Аннотация

Введение: Мужчины, имеющие секс с мужчинами (МСМ), такие люди стали группой с самыми высокими темпами роста эпидемии ВИЧа в Китае. Поскольку многие китайские МСМ проводят самотестирование на ВИЧ, мы стремились определить уровень ВИЧ по обращению за медицинской помощью после самотестирования, изучить характеристики “искатели” по сравнению с “не-искателей” и исследовать факторы, связанные с поведением ухода за выздоравливающим ВИЧ.

Методы исследования: В поперечном сечении было использованно дизайн-исследование и онлайн-опрос был проведен в Пекине Китая в 2016 году, среди пользователей по одному популярному сетевому приложению о китайских голубых мужчинах для смарт-телефонов. Chi-square тест был использован для сравнения характеристик тех, кто стремился к уходу в связи с ВИЧ (“искатели”) и те, кто не (“не-искатель”). Однофакторный и многофакторный анализ логистической регрессии были проведены для оценки факторов, связанных с поиском ухода в связи с ВИЧ.

Результаты исследования: Среди 21,785 человек, которые были экранированны, 2 383 участников (10.9%) были включены в исследовании. В общей сложности 380 участников

(15.9%) сообщили уход за выздоравливающим ВИЧ после заражения ВИЧ самотестирования при 2 003 (84.1%) не сообщили. Незнание “периода окна” (скорректированное отношение шансов [СОШ] = 0.68, 95% доверительных интервалов [95% *CI*] = 0.47 – 0.97, *P* = 0,04) были связаны с уменьшенным коэффициентом по обращению ухода при ВИЧ / СПИДе, в то время как низкий ежемесячный доход (A*OR* = 1.29, 95% *CI* = 1,03 – 1.62, *P* = 0,03) и получения самотестирования на ВИЧ наборы медицинских учреждений (A*OR*=2.40, 95% *CI*=1.81–3.17, *P* < 0,001), и неправительственные организации (A*OR*=2.44, 95% *CI* = 1.79 – 3.34, *P* < 0,001) были связаны с повышенным коэффициентом по обращающию за лечением ВИЧ. Среди тех, кто обращался за лечением ВИЧ-инфекции, подавляющее большинство (92.4%) имело нереактивное самотестирование на результаты ВИЧ. Только 29 из 265 с реактивными, неопределенными или неизвестными результатами стремились к уходу в связи с ВИЧ.
Заключеине: Мы нашли очень низкий уровень по обращению за уходом за выздоравливающим ВИЧ в нашей выборке городского китайской МСМ. Наблюдение показанно, что большинство больных с реактивным, неопределенными или неизвестными результатами и это значит: уход ВИЧ не является причиной для беспокойства. Этим людям следует уделять больше внимания и помогть войти в помощи каскада. Наши выводы подчеркивают, что мероприятия, направленные на улучшение связи с лечением после самотестирование на ВИЧ крайне необходимы. Тем не менее, дальнейшие исследования необходимы для того, чтобы сообщить разработки и осуществления будущих мероприятий, направленные на стимулирование поведения ухода за выздоравливающим ВИЧ.

Translated from English version into French by Hao-Qi Zhang

El comportamiento de búsqueda de cuidado para el VIH después de la auto-prueba del VIH entre hombres que tienen relaciones sexuales con hombres en Beijing, China: un estudio transversal

Xian-Long Ren, Zun-You Wu, Guo-Dong Mi, Jennifer M. McGoogan, Ke-Ming Rou[1], Yan Zhao and Nan-Ci Zhang

Abstracto

Trasfondo: Los hombres que tienen relaciones sexuales con hombres (MSM, por sus siglas en inglés) se han convertido en el grupo con la epidemia de VIH de más rápido crecimiento en China. Dado que muchos MSM chinos están llevando a cabo la auto-prueba del VIH, nuestro objetivo fue determinar la tasa de atención y cuidado del VIH después de la auto-prueba, examinar las características de los "buscadores" en comparación con "no buscadores", y explorar los factores asociados con el comportamiento de búsqueda de atención para el VIH.
Métodos: Se utilizó un diseño de estudio transversal y se realizó una encuesta en línea en Beijing, China, en el 2016, entre los usuarios de una aplicación china para teléfonos inteligentes diseñada para la socialización de homosexuales. Se usó la prueba del Chi cuadrado para comparar las características de los que buscaron el cuidado del VIH ("buscadores") y los que

no lo hicieron ("no buscadores"). Se realizaron análisis de regresión logística univariante y multivariante para evaluar los factores asociados con la búsqueda del cuidado del VIH.

Resultados: Entre los 21, 785 seleccionados, 2, 383 participantes (10.9%) fueron incluidos en el estudio. Un total de 380 participantes (15.9%) informaron solicitar atención para el VIH después de la auto-prueba del VIH, mientras que 2, 003 (84.1%) no lo hicieron. La falta de conocimiento del "período de ventana" (razón ajustada a probabilidad [AOR] = 0.68, intervalo de confianza del 95% [IC del 95%] = 0.47-0.97, P = 0.04) se asoció con una menor probabilidad de buscar atención, mientras que un ingreso mensual bajo (AOR = 1.29; IC del 95%: 1.03-1.62; P = 0.03) y la obtención de kits de autodiagnóstico del VIH en los centros de salud (AOR = 2.40, IC del 95% = 1.81 – 3.17, P <0.001) y organizaciones no gubernamentales (AOR = 2.44, IC del 95% = 1.79-3.34, P <0.001) se asoció con mayores probabilidades de buscar el cuidado del VIH. Entre los que buscaron el cuidado del VIH, una gran mayoría (92.4%) tuvieron resultados de auto-prueba de VIH no reactivos. Sólo 29 de 265 con resultados reactivos, inciertos o desconocidos buscaron el cuidado del VIH.

Conclusiones: Encontramos una tasa muy baja de búsqueda de cuidado para el VIH entre nuestra muestra de MSM chinos urbanos. La observación de que la mayoría con resultados reactivos, inciertos o desconocidos no buscó el cuidado del VIH es motivo de preocupación. A estas personas se les debería prestar más atención y ayudar a entrar en la cascada de cuidado médico. Nuestros resultados apuntan que las intervenciones destinadas a mejorar la vinculación a la atención médica después de la auto-prueba del VIH se necesitan con urgencia. Sin embargo, se necesita más estudio para sugerir el diseño y la implementación de futuras intervenciones que apunten a fomentar el comportamiento de búsqueda del cuidado médico para el VIH.

Translated from English version into French by Laura C Vicente Rodriguez
